# Free‐living associations between resting heart rate parameters and cardiometabolic health in adults

**DOI:** 10.14814/phy2.71008

**Published:** 2026-07-06

**Authors:** Roberto A. González‐Fimbres, Claudia S. Cuevas‐Castro, María G. Ramírez‐Siqueiros, Teresita Valencia‐Falcón, Andrew Flatt

**Affiliations:** ^1^ Licenciatura en Entrenamiento Deportivo, Unidad Académica Hermosillo Universidad Estatal de Sonora Hermosillo Sonora México; ^2^ Health Sciences and Kinesiology, Biodynamics and Human Performance Center Georgia Southern University Savannah Georgia USA

**Keywords:** autonomic function, exercise physiology, metabolic health, physiological monitoring, RMSSD, wearable technology

## Abstract

This study examined associations between multi‐day ultra‐short resting heart rate (HR) parameters and cardiometabolic health under free‐living conditions. Sixty‐seven adults (18–59 years, 53% male) completed seven consecutive days of seated 1‐min RR interval recordings after waking using a validated chest strap monitor. Log‐transformed root mean square of successive differences (LnRMSSD_M_) and resting HR (HR_M_) were averaged across days and standardized. Cardiometabolic health status was defined as the number of metabolic syndrome (MetS) components present. Associations were assessed using Poisson regression adjusted for age and sex, with additional models including HR_M_ and HR‐adjusted LnRMSSD. Higher LnRMSSD_M_ was associated with fewer MetS components (IRR = 0.79, 95% CI: 0.64–0.97), whereas higher HR_M_ was associated with greater metabolic burden (IRR = 1.40, 95% CI: 1.10–1.77). When both variables were included, the association between LnRMSSD_M_ and MetS was attenuated and no longer significant (IRR = 0.93, 95% CI: 0.72–1.22), while HR_M_ remained significant (IRR = 1.33, 95% CI: 1.00–1.77). Resting HR parameters obtained through accessible devices were associated with cardiometabolic health, with resting HR accounting for much of the cross‐sectional information captured by LnRMSSD. Remote cardiac monitoring may support early cardiometabolic risk identification and longitudinal health surveillance.

## 1 INTRODUCTION

Heart rate variability (HRV), particularly vagally mediated indices such as the root mean square of successive differences (RMSSD), is widely used in sport and exercise science as a non‐invasive marker of autonomic function and physiological status (González‐Fimbres et al., 2021; Jarczok et al., 2013; O'Connor et al., 2024). Its practical utility has increased substantially in recent years due to the widespread adoption of wearable devices and smartphone‐based applications, which enable frequent and ecologically valid monitoring of cardiovascular dynamics in both athletic and general populations (Dobbs et al., 2019; Grosicki et al., 2022; Nakamura et al., 2016; Vondrasek et al., 2023). Daily HRV assessment, especially when averaged across multiple days, has been proposed as a reliable approach to track short‐term physiological fluctuations and longer‐term adaptations in response to training, lifestyle, and health‐related factors (Bellenger et al., 2022; Vila et al., 2019). Consequently, ultra‐short HRV recordings have emerged as a scalable and accessible tool for monitoring cardiac autonomic status under real‐world conditions.

Beyond its application in performance settings, HRV has also been linked to broader indicators of physiological health. Metabolic syndrome (MetS), defined as a clustering of cardiometabolic abnormalities including central adiposity, dyslipidemia, hypertension, and impaired glucose regulation, represents a useful index of systemic physiological strain and health status (Kim et al., 2021; Subhashini et al., 2020). Reduced HRV has been consistently associated with adverse metabolic profiles and increased cardiometabolic burden, suggesting that vagally mediated HRV may reflect the integrative impact of metabolic dysfunction on autonomic regulation (Jarczok et al., 2013; Stuckey et al., 2014; Williams et al., 2019). In this context, HRV has been proposed as a non‐invasive marker capable of capturing early alterations in physiological homeostasis across a continuum of health and disease. Accordingly, examining associations between remote HRV tracking and cardiometabolic status in free‐living conditions is an important next step toward developing scalable detection, intervention, and monitoring strategies.

An important methodological and physiological limitation in the interpretation of HRV is its intrinsic dependence on underlying heart rate (HR) dynamics. Due to the nonlinear relationship between HR and R–R interval variability, HRV may increase as HR decreases even in the absence of changes in autonomic modulation (Sacha, 2013, 2014). Experimental and theoretical work further suggests that HRV indices are strongly influenced by the biophysical properties of sinoatrial node activity, reflecting both mathematical coupling and physiological interdependence between HR and variability (Monfredi et al., 2014). In addition, resting HR is itself modulated by the balance between parasympathetic withdrawal and sympathetic activation, and elevated HR has been associated with increased cardiometabolic risk and sympathetic overdrive (Dell'Oro et al., 2024; Grassi et al., 2025). Accordingly, the overlap between HR and HRV may represent a shared expression of autonomic regulation rather than independent physiological constructs, raising questions about the extent to which HRV provides unique information beyond HR (de Geus et al., 2018). Despite growing interest in ultra‐short HRV monitoring, relatively few studies have examined whether multi‐day HRV measures provide information that is independent from HR when assessed under free‐living conditions using consumer‐accessible technologies. Therefore, this study examined associations between multi‐day resting cardiac autonomic parameters (HR and RMSSD) and the accumulation of MetS components and evaluated whether RMSSD‐related associations persisted after accounting for resting HR dynamics.

## 2 MATERIALS AND METHODS

### 2.1 Design

This cross‐sectional study consisted of a 7‐day observational period of HRV monitoring under free‐living conditions, followed by a single laboratory visit to assess cardiometabolic health and fitness. The protocol was designed to reflect real‐world monitoring conditions using consumer‐accessible wearable technology. Participants were instructed to maintain their habitual lifestyle behaviors, including physical activity, diet, and sleep–wake patterns, to enhance ecological validity.

Participants returned to the laboratory within 5 days of completing the observation period for a comprehensive health evaluation. All testing was conducted in the morning (07:00–09:00). Participants arrived fasted after abstaining from food and beverages for at least 12 h and were instructed to avoid vigorous physical activity, caffeine, and alcohol for 24 h before testing. Assessments were performed in a standardized order: anthropometry, waist circumference, resting blood pressure, capillary blood sampling, body composition, handgrip strength, and cardiorespiratory fitness.

### 2.2 Participants

Sixty‐seven adults (18–59 years) were recruited from the local community using convenience sampling. Participation was voluntary and required the ability to comply with study procedures and access to a compatible smartphone for HRV monitoring. Participants were recreationally active adults representing a heterogeneous range of fitness and physiological status. Individuals with medical conditions or treatments known to influence autonomic regulation, metabolic function, or exercise responses were excluded. Participants were removed from the final analysis if HRV recordings were incomplete or contained artifacts that prevented reliable HRV quantification. The study protocol was approved by the Research Ethics Committee of the University of Juárez (CONBIOETICA‐10‐CEI001‐20220131). All participants provided written informed consent prior to participation, and procedures were conducted in accordance with the Declaration of Helsinki.

### 2.3 Procedures

#### 2.3.1 Heart rate variability

To record HRV, the Polar H10 heart rate monitor with a sampling frequency of 1000 Hz (Polar Electro Oy, Kempele, Finland) was used, which has been suggested as a suitable instrument for collecting HRV data in field‐based settings (Blalock et al., 2026). Recordings were performed immediately after awakening. Participants were instructed to place the transmitter next to their bed before going to sleep to facilitate placement the following morning with minimal disturbance. Before placement, they were asked to moisten the electrodes of the chest strap and position it around the chest in direct contact with the skin. Subsequently, participants assumed a seated position in a back‐supported chair; breathing was not controlled to preserve ecological validity. HRV recordings were obtained using the Elite HRV application (Version 5.5.8 # 79, Asheville, NC, USA) for R–R interval data storage (Vondrasek et al., 2023). Ultra‐short recording periods were one‐minute in duration, preceded by a one‐minute stabilization period; this has been shown to provide valid estimates of RMSSD under resting conditions (Grosicki et al., 2022). Participants performed daily recordings over 1 week. This protocol was selected to reflect common applied monitoring practices used in sport and exercise settings. After the completion of the recording week, data were exported from the Elite HRV application to a computer for subsequent analysis using Kubios HRV Scientific software (Version 4.2.0, University of Eastern Finland, Kuopio, Finland). All R‐R interval recordings were visually inspected for signal quality and artifact presence. Recordings with excessive artifacts (>5% corrected beats) or poor signal quality were excluded from further analysis. For the remaining recordings, artifact correction was performed using the Kubios automatic artifact correction filter set at the lowest threshold (Flatt & Esco, 2015). A total of 108 participants were initially recruited. Of these, 35 participants were excluded because they did not provide the minimum number of valid HRV recordings required for analysis, and 6 participants were excluded because signal quality issues or excessive artifacts prevented reliable HRV quantification. Consequently, 67 participants were included in the final analysis. Consistent with a prior investigation demonstrating that weekly LnRMSSD values derived from at least 5 recordings provide very good agreement with 7‐day averages (Flatt & Esco, 2015), participants were required to provide a minimum of 5 valid recordings out of the 7 scheduled measurements for inclusion (Medeiros et al., 2021). The mean LnRMSSD (LnRMSSD_M_), was calculated for each valid daily sample and then averaged across available recordings to derive the weekly mean value (Grosicki et al., 2022).

#### 2.3.2 MetS components

The lipid profile was assessed using a lipid analyzer (Mission Cholesterol Monitoring System; ACON Laboratories Inc., San Diego, CA, USA) with a 3‐in‐1 reagent panel for the determination of total cholesterol, HDL cholesterol, and triglycerides. Capillary blood was obtained via finger puncture using a lancing device, and the blood sample was collected with a micropipette and applied to the reagent strip. Results were obtained following the procedure specified by the device manufacturer. Blood glucose levels were measured using the Accutrend Plus analyzer (Roche Diagnostics, Mannheim, Germany) with Accutrend glucose test strips (REF 11447475). Capillary blood collection followed the same procedure described above. Waist circumference was measured according to the International Society for the Advancement of Kinanthropometry (ISAK) protocol. Blood pressure was measured using an automatic wrist blood pressure monitor (Rossmax, Taipei, Taiwan) that has previously been validated (Zeng et al., 2012). Measurements were obtained with participants seated and resting for at least 5 min in a quiet environment, with the back supported, feet flat on the floor, and the arm positioned so that the wrist cuff was maintained at heart level. Participants were instructed to remain still and avoid talking during the measurement. Two readings were taken with an interval of approximately 1 min, and the average value was used for analysis.

#### 2.3.3 Body composition

Body composition was assessed using dual‐energy X‐ray absorptiometry (DXA) for whole‐body analysis with a PRIMUS/OSTEOSYS system (Osteosys Co., Ltd. Seoul, South Korea). DXA is widely considered a reference method for assessing body composition due to its high precision and validity in quantifying total and regional adiposity, as well as lean tissue distribution in adult populations. In addition to total body composition, DXA provides estimates of visceral adipose tissue (VAT). Participants were scanned in a fasted state, wearing light clothing, without metallic objects, and barefoot. During the assessment, participants were positioned supine on the scanning table and instructed to maintain a standardized anatomical posture throughout the procedure. Whole‐body scans lasted approximately 8 min and were performed using a low radiation dose (≈5.08 μGy), in accordance with international safety standards (Skalsky et al., 2012). The DXA software automatically generated body composition reports, from which adiposity‐ and cardiometabolic‐related variables were extracted, including total body fat percentage (BF%), lean body mass (LBM), visceral adipose tissue mass, and VAT area. DXA‐derived measures were exported for statistical analysis and used as objective indicators of body composition and regional adiposity within the analytical models.

#### 2.3.4 Physical fitness

Cardiorespiratory fitness was evaluated using a standardized 1‐mile field test based on the Rockport Fitness Walking Test protocol (Dolgener et al., 1994). Participants were instructed to complete a fixed distance of 1 mile (1609 m) on a measured course as quickly as possible while maintaining a steady, continuous pace. To ensure inclusivity and methodological consistency across participants with heterogeneous fitness and functional capacities, individuals were allowed to complete the test either by fast walking or running, depending on their physical ability. This approach enabled the use of a single assessment method for participants who were able to run and those who were limited to walking, thereby avoiding the application of different tests or equations across subgroups. Upon completion of the 1‐mile distance, HR was recorded with the Polar H10 monitor using the Polar Beat application (Version 3.5.12, V. Polar Electro, Kempele, Finlad), and total completion time was documented. These variables, together with sex, age, and body mass, were used to estimate maximal oxygen uptake (VO_2_max) using a Rockport‐based prediction equation (Dolgener et al., 1994). The use of this equation was justified by its reliance on both external workload (distance and time) and internal physiological response (end‐exercise HR), which together provide a robust field‐based estimate of cardiorespiratory fitness. Allowing higher‐fit participants to run reduced potential ceiling effects associated with walking‐only protocols and enhanced the sensitivity of the test to detect interindividual differences in aerobic capacity. The resulting VO_2_max values were treated as estimates suitable for comparative and correlational analyses within the study sample. Handgrip strength was assessed using an electronic handgrip dynamometer (Constant, model 14,192‐709E) following ACSM guidelines. Participants stood with arms extended alongside the body and a neutral wrist, squeezing maximally for 3–5 s. Two to three trials per hand were performed with 60 s rest. The highest value recorded (lb) was used for analysis.

### 2.4 Statistical analysis

Continuous variables were inspected for normality using the Shapiro–Wilk test and visual examination of distribution plots. Descriptive data are presented as mean ± standard deviation for continuous variables and as frequencies and percentages for categorical variables. Sex differences in the prevalence of MetS components were evaluated using contingency tables and Fisher's exact test due to small cell counts. Associations between LnRMSSD_M_ and continuous metabolic variables were explored using Pearson correlation coefficients. Before multivariable modeling, LnRMSSD_M_ was standardized (*z*‐score) to facilitate interpretation of effect estimates. Collinearity among candidate predictors was assessed using correlation matrices; body composition variables and cardiorespiratory fitness showed strong intercorrelations and were therefore not included simultaneously in the same multivariable models. The association between HRV and the number of MetS components was examined using Poisson regression with robust standard errors, adjusted for age and sex. Results are reported as incidence rate ratios (IRR) with 95% confidence intervals (95% CI). Additional models incorporated resting HR_M_ and HR‐corrected HRV, derived using a residualization approach in which standardized LnRMSSD_M_ was regressed on standardized HR_M_, and the residuals were retained as HR‐corrected HRV. Binary logistic regression models were used to evaluate whether LnRMSSD_M_ predicted the presence of individual metabolic alterations (low HDL cholesterol, elevated glucose, triglycerides, blood pressure, and waist circumference) as well as metabolic syndrome (≥3 criteria). Models were adjusted for age and sex, and results are presented as odds ratios (OR) with 95% CI. The discriminative capacity of logistic models was assessed using receiver operating characteristic (ROC) curves and the area under the curve (AUC). Statistical significance was set at *p* < 0.05. All analyses were performed using SPSS (version 25, IBM Corp., Chicago, IL, USA).

## 3 RESULTS

Data from 67 out of 108 participants (36 men and 31 women) were included in the analysis. Comparisons between those who were included (*n* = 67) and those who were excluded due to incomplete or poor‐quality HRV recordings (*n* = 41) revealed no statistically significant sex‐, cardiometabolic‐, anthropometric‐, or fitness‐related differences (all *p* > 0.12). Age tended to be higher in excluded participants, although this did not reach statistical significance (*p* = 0.056).

Included participants displayed substantial variability in anthropometric, metabolic, and physiological characteristics. Table 1 summarizes participant characteristics according to the number of MetS components present. A progressive increase in body weight, body mass index, waist circumference, triglycerides, glucose levels, and visceral adipose tissue was observed as the number of MetS components increased. Conversely, HDL cholesterol and estimated cardiorespiratory fitness tended to decrease across increasing MetS categories. Descriptive data indicated a progressive decline in LnRMSSD_M_ with increasing metabolic burden.

**TABLE 1:** Participant characteristics by the number of metabolic syndrome components.

MetS factors (*n*)	0 (23)	1 (11)	2 (18)	3 (5)	4 (8)	5 (2)
Age (years)	34.83 ± 11.45	42.27 ± 10.09	41.61 ± 8.15	37.4 ± 13.35	45.25 ± 5.31	37.00 ± 2.82
Weight (kg)	66.52 ± 13.64	73.31 ± 14.36	84.54 ± 22.15	93.92 ± 22.91	104.2 ± 29.12	141.1 ± 15.7
BMI (kg·m^−2^)	23.26 ± 2.77	24.95 ± 3.48	29.97 ± 6.39	30.23 ± 5.99	35.54 ± 7.51	40.99 ± 4.25
Waist circ. (cm)	73.7 ± 9.35	82.14 ± 15.47	98.29 ± 28.56	101.4 ± 17.56	110.2 ± 16.87	127 ± 16.97
Total Cholesterol (mg/dL)	186 ± 60.65	192.6 ± 69.36	185.8 ± 44.65	206.4 ± 70.61	213.4 ± 53.33	154.5 ± 58.69
HDL (mg/dL)	63.74 ± 15.31	56.18 ± 17.82	50.33 ± 17.17	32.6 ± 9.12	35.13 ± 6.55	22 ± 4.24
Triglycerides (mg/dL)	98.22 ± 21.44	111.1 ± 51.59	136.3 ± 73.47	182.4 ± 84.28	195.9 ± 64.57	198 ± 57.98
LDL (mg/dL)	103 ± 52.39	122.7 ± 55.93	112.4 ± 40.19	142.4 ± 47.8	139.8 ± 43.11	93.5 ± 65.76
Glucose (mg/dL)	93.5 ± 3.85	96 ± 9.25	105.3 ± 9.59	101.8 ± 7.79	122.4 ± 20.88	112.5 ± 13.44
Systolic BP (mmHg)	112.2 ± 11.46	117.2 ± 7.58	120.6 ± 11.93	126.2 ± 11.97	125.6 ± 9.56	135 ± 7.07
Diastolic BP (mmHg)	68.26 ± 8.76	75.36 ± 5.90	75.94 ± 11.68	84.4 ± 11.55	82.5 ± 5.95	90 ± 0
Grip Strength (lb)	77.41 ± 27.55	78.43 ± 24.69	83.5 ± 26.13	105.3 ± 21.63	95.77 ± 26.19	141.6 ± 7.63
VO_2_max (mL/kg/min)	44.76 ± 6.56	46.24 ± 6.55	40.29 ± 6.88	41.25 ± 6.67	35.15 ± 3.17	32.69 ± 2.79
BF %	21.9 ± 8.37	25.06 ± 7.04	30.74 ± 8.80	31.42 ± 5.02	37.46 ± 5.65	33.65 ± 4.31
VAT (g)	254.4 ± 242.1	520.7 ± 342.9	810.3 ± 464.4	1055 ± 378.9	1443 ± 470.1	1534 ± 631.4
VAT area (cm^2^)	53.96 ± 49.52	135.1 ± 103.6	168.7 ± 96.65	219.6 ± 78.86	300.1 ± 97.57	319 ± 131.5
LBM (kg)	49.97 ± 11.67	52.21 ± 8.68	54.76 ± 12.01	60.5 ± 10.93	61.03 ± 15.02	85.05 ± 0.35
LBM Index (kg/m^2^)	17.44 ± 2.63	17.75 ± 1.68	19.35 ± 2.42	19.52 ± 2.45	20.75 ± 3.12	24.72 ± 0.29
LnRMSSD_M_ (ms)	3.749 ± 0.55	3.723 ± 0.56	3.368 ± 0.39	3.445 ± 0.66	3.393 ± 0.43	3.466 ± 0.88
HR_M_ (bpm)	62.67 ± 12.04	61.45 ± 7.87	66.53 ± 8.55	64.25 ± 8.74	72.11 ± 6.53	55.98 ± 7.02

*Note*: Values are presented as mean ± SD. All laboratory assessments were performed following a minimum of 12 h overnight fast.

Abbreviations: BF%, percentage of body fat; BMI, body mass index; BP, blood pressure; HDL, High‐Density Lipoprotein; HR_M_, 7‐day resting heart rate meanLBM, lean body mass; LDL, Low‐Density Lipoprotein; LnRMSSD_M_, 7‐day log‐transformed root mean square of successive differences mean; MetS, metabolic syndrome; VAT, visceral adipose tissue.

The prevalence of metabolic alterations differed between sexes (Table 2). Men exhibited a significantly higher prevalence of low HDL cholesterol and elevated waist circumference compared with women (*p* < 0.05). The prevalence of metabolic syndrome (≥3 criteria) was also significantly higher in men than in women (*p* < 0.05). No statistically significant sex differences were observed for elevated triglycerides, glucose, or blood pressure.

**TABLE 2:** Prevalence of metabolic syndrome components according to sex.

Metabolic component	Men (%)	Women (%)	Fisher *p*‐value
Low HDL cholesterol	50.0	19.4	**0.01**
Elevated triglycerides	33.3	19.4	0.27
Elevated glucose	50.0	41.9	0.63
Elevated blood pressure	19.4	12.9	0.53
Elevated waist circumference	41.7	16.1	**0.03**
Metabolic syndrome (≥3 criteria)	33.3	9.7	**0.04**

*Note*: Bold values indicate statistical significance (*p* < 0.05).

Correlation analyses revealed modest but significant associations between LnRMSSD_M_ and several cardiometabolic variables. LnRMSSD_M_ correlated positively with estimated VO_2_max (*r* = 0.34, *p* < 0.01) and negatively with triglycerides (*r* = −0.28, *p* < 0.05), body fat percentage (*r* = −0.31, *p* < 0.05), and VAT (*r* = −0.25, *p* < 0.05). In contrast, correlations between LnRMSSD_M_ and traditional anthropometric indicators such as body weight, BMI, and waist circumference were weak and not statistically significant (*r* < 0.15, *p* > 0.05).

Correlation matrices revealed substantial collinearity among VO_2_max, BMI, waist circumference, body fat percentage, VAT, and VAT area (*r* = 0.66–0.81). Consequently, these variables were not included simultaneously in multivariable models.

Poisson regression analysis showed that higher LnRMSSD_M_ was significantly associated with a lower number of MetS components after adjustment for age and sex. An increase of one standard deviation in LnRMSSD_M_ (mean = 3.54 ± 0.55), corresponding approximately to an increase in RMSSD from 34.6 ms to 60.2 ms, was associated with an approximate 21% reduction in the expected number of MetS component count per 0.55‐unit increase in LnRMSSD_M_ (IRR = 0.79; 95% CI: 0.64–0.97; *p* = 0.03). Age (IRR = 1.02; *p* < 0.05) and male sex (IRR = 1.74; *p* < 0.05) were positively associated with the accumulation of metabolic risk factors. In contrast, standardized resting HR_M_ was positively associated with MetS components, with each 1‐SD increase (approximately 8.5 bpm) corresponding to a 39.7% increase (IRR = 1.40; 95% CI: 1.10–1.77; *p* < 0.01). When both variables were included, the association of LnRMSSD_M_ was attenuated and no longer significant (IRR = 0.93; 95% CI: 0.72–1.22; *p* = 0.61), whereas resting HR_M_ remained the dominant predictor (IRR = 1.33; 95% CI: 1.00–1.77; *p* = 0.05), corresponding to a 33.0% increase per 1‐SD. HR‐corrected HRV was not associated with MetS components (IRR = 0.90; 95% CI: 0.69–1.17; *p* = 0.42).

Table 3 shows the logistic regression models used to evaluate HRV as a predictor of individual dichotomous metabolic alterations. After adjustment for age and sex, LnRMSSD_M_ remained associated only with reduced HDL cholesterol, showing a strong inverse relationship and moderate discriminative performance (AUC = 0.75, 95% CI OR = 0.078–0.789). No significant associations were observed for elevated glucose, triglycerides, blood pressure, or waist circumference. Finally, analyses showed that LnRMSSD_M_ was not independently associated with the presence of MetS as a composite clinical condition after adjustment for age and sex. Nevertheless, the model showed moderate classification ability (AUC = 0.72, 95% CI OR = 0.149–1.658).

**TABLE 3:** Logistic regression models evaluating mean LnRMSSD_M_ as a predictor of dichotomous metabolic alterations (adjusted for age and sex).

Metabolic variables	B (LnRMSSD)	SE	Wald *χ* ^2^	*p*‐value	OR = Exp (B)	95% CI OR	AUC
Low HDL cholesterol	−1.396	0.593	5.537	**0.02**	0.248	0.078–0.789	0.749
Elevated glucose	−0.604	0.523	1.335	0.25	0.547	0.196–1.525	—
Elevated triglycerides	−0.577	0.549	1.104	0.29	0.562	0.192–1.646	—
Elevated blood pressure	−0.989	0.688	2.069	0.15	0.372	0.097–1.425	—
Elevated waist circumference	−0.352	0.565	0.388	0.53	0.704	0.233–2.128	0.731
Metabolic syndrome (≥3 criteria)	−0.698	0.614	1.293	0.26	0.497	0.149–1.658	0.720

*Note*: Bold values indicate statistical significance (*p* < 0.05).

## 4 DISCUSSION

The present study demonstrates that ultra‐short post‐waking LnRMSSD_M_ is inversely associated with cardiometabolic health status after adjustment for age and sex. Specifically, each 1‐SD increase in LnRMSSD_M_ was associated with a ~21% reduction in the expected number of MetS components; however, this relationship appears to be strongly influenced by underlying HR dynamics. These findings indicate that ultra‐short resting HR and LnRMSSD reflect overlapping aspects of autonomic regulation, with resting HR accounting for a substantial portion of the cross‐sectional association observed between HRV and cardiometabolic health under real‐world monitoring conditions.

This pattern is consistent with the concept of HR dependence of HRV, whereby indices such as RMSSD are influenced not only by autonomic modulation but also by underlying cardiac cycle dynamics (Sacha, 2013, 2014). According to contemporary models of autonomic cardiovascular regulation, resting HR is more closely related to the tonic level of autonomic neural activity acting on the sinoatrial node, whereas HRV magnitude reflects beat‐to‐beat fluctuations around that tonic level and therefore the modulation of autonomic inputs (La Rovere et al., 2020). Due to the nonlinear relationship between HR and R–R interval variability, reductions in HR inherently increase variability even in the absence of changes in vagal activity (Monfredi et al., 2014). Consequently, part of the association between HRV and health outcomes may reflect shared mathematical and physiological variance with HR, rather than exclusively independent autonomic regulation (de Geus et al., 2018). Importantly, this overlap is not purely methodological. Resting HR reflects both vagal withdrawal and sympathetic activation, and elevated HR has been associated with sympathetic overdrive and increased cardiometabolic risk, particularly at values exceeding 70–80 beats·min^−1^ (Dell'Oro et al., 2024; Grassi et al., 2025). Thus, the stronger association observed for HR_M_ may indicate that metabolic burden is more closely linked to chronic alterations in autonomic tone, whereas LnRMSSD_M_ captures a more specific, but partially overlapping, component of autonomic modulation. In other words, individuals with greater metabolic burden appear to be characterized primarily by a shift in the autonomic set point (reflected by elevated HR), while beat‐to‐beat variability around this set point (RMSSD) provides limited additional explanatory value in this cross‐sectional context.

The present findings extend previous work demonstrating associations between HRV and metabolic health. Consistent with prior studies, LnRMSSD_M_ was modestly associated with VO_2_max and inversely related to triglycerides, adiposity, and visceral fat (Jarczok et al., 2013; Velasquez et al., 2018). Similarly, large population‐based studies have reported progressive reductions in HRV with increasing MetS components (Azulay et al., 2022). However, unlike Grosicki et al. (2022), who found that associations between HRV and health markers were attenuated after adjustment for cardiorespiratory fitness, the present study suggests that resting HR may play a more prominent explanatory role in heterogeneous populations characterized by greater metabolic impairment. This discrepancy likely reflects differences in sample characteristics and the relative contribution of fitness versus adiposity and autonomic dysregulation.

A key contribution of this study lies in conceptualizing HRV as a marker of cumulative metabolic burden rather than a discriminator of isolated abnormalities. While LnRMSSD_M_ showed limited associations with individual dichotomous metabolic components, remaining significantly associated only with reduced HDL cholesterol, it was consistently related to the overall clustering of risk factors. This finding underscores the limitation of dichotomizing continuous physiological processes and supports the use of count‐based or gradient‐based models to better capture early systemic dysregulation.

From a mechanistic perspective, progressive visceral adiposity, insulin resistance, oxidative stress, and low‐grade inflammation contribute to cumulative autonomic dysregulation (Stuckey et al., 2014; Williams et al., 2019). As these processes intensify, parasympathetic withdrawal likely reflects not only altered autonomic signaling but also progressive disruption of cardiovagal pathways, consistent with early neuropathic processes affecting the vagus nerve. The observed association between LnRMSSD_M_ and metabolic burden is therefore physiologically consistent; however, its attenuation after HR adjustment indicates that part of this signal is influenced by shared physiological and mathematical coupling with HR (Monfredi et al., 2014). These findings reinforce the importance of interpreting HRV within the context of underlying HR dynamics.

The moderate discriminative performance observed in logistic models (AUC≈0.72–0.75) further supports the interpretation of ultra‐short HRV as a tool for risk stratification rather than a standalone diagnosis. In practical terms, its utility may lie in longitudinal monitoring, where repeated measurements can detect early deviations from baseline before clinical thresholds are reached.

Sex‐related differences in metabolic risk were also evident, with men exhibiting a higher prevalence of abdominal obesity, low HDL cholesterol, and MetS. These findings are consistent with established sexual dimorphism in adipose tissue distribution and cardiometabolic regulation, where greater visceral fat accumulation in men is associated with increased metabolic and autonomic dysfunction (Jawwa et al., 2022; Milić et al., 2014). Such differences may partly explain the observed variation in HRV across MetS categories.

The use of a non‐probabilistic convenience sample and the relatively modest sample size are considered limitations to the generalizability of the results; also, this may reduce statistical power to detect associations with individual metabolic abnormalities. Moreover, the lower prevalence of MetS among women compared with men may have introduced a degree of sex‐related sampling imbalance, potentially influencing statistical power and the generalizability of the findings. Additionally, lifestyle variables that influence autonomic regulation, including habitual physical activity, sleep quality, and dietary patterns, were not formally controlled in the statistical models. Likewise, the menstrual cycle phase was not assessed in female participants. Given that hormonal fluctuations throughout the menstrual cycle can affect HRV, resting HR, and metabolic outcomes, the absence of this information may have introduced unmeasured variability into the analyses. Furthermore, interpretation of RMSSD as a cardiometabolic risk indicator may be limited by its moderate heritability, as genetically predicted HRV has not been associated with mortality despite robust phenotypic associations (Tegegne et al., 2023). Also, the absence of respiratory frequency measurements prevented the assessment of high‐frequency spectral power, limiting the ability to evaluate more specific indices of vagal cardiac control beyond LnRMSSD (Menuet et al., 2025). Finally, resting LnRMSSD captures a limited, tonic component of cardiovagal control, whereas evidence from standardized cardiovagal reflex tests indicates that dynamic HR responses can reveal early cardiometabolic‐related autonomic dysfunction before resting HR abnormalities emerge (Ewing & Clarke, 1982). Despite these limitations, the study presents several strengths, including the use of multi‐day HRV averaging, standardized morning recordings, objective body composition assessment using DXA, and the modeling of metabolic burden as a cumulative outcome rather than relying solely on dichotomous diagnostic thresholds.

## 5 CONCLUSIONS

Multi‐day ultra‐short HRV, assessed via LnRMSSD_M_, is associated with cardiometabolic health status when conceptualized as a continuum; however, this relationship appears to be partly driven by underlying HR dynamics. The attenuation of LnRMSSD_M_ after accounting for resting HR_M_ suggests that both metrics reflect overlapping aspects of autonomic regulation when assessed cross‐sectionally rather than independent physiological processes. Our findings extend cardiac autonomic–cardiometabolic associations previously demonstrated under controlled laboratory conditions to remote, free‐living monitoring with accessible technologies. Accordingly, remote monitoring of cardiac HR parameters may facilitate early identification of cardiometabolic risk and related cardiac autonomic alterations, while supporting intervention opportunities and longitudinal monitoring.

## AUTHOR CONTRIBUTIONS

**Roberto A. González‐Fimbres:** Conceptualization; data curation; formal analysis; funding acquisition; investigation; methodology; project administration. **Claudia S. Cuevas‐Castro:** Formal analysis; investigation. **María G. Ramírez‐Siqueiros:** Conceptualization; formal analysis; investigation; methodology. **Teresita Valencia‐Falcón:** Conceptualization; investigation; project administration. **Andrew Flatt:** Conceptualization; data curation; formal analysis; methodology.

## FUNDING INFORMATION

The Universidad Estatal de Sonora supported this work through an internal research funding program under Grant number UES‐PII‐23‐UAH‐LED‐01.

## CONFLICT OF INTEREST STATEMENT

The authors report no financial or personal relationships with other people or organizations that could inappropriately influence or bias their work. The authors report no conflict of interest.

## ETHICS STATEMENT

The study protocol was approved by the Research Ethics Committee of the University of Juárez, Estado de Durango (CONBIOETICA‐10‐CEI001‐20220131). All participants provided written informed consent prior to participation. All procedures performed in this study involving human participants were conducted in accordance with the ethical standards of the institutional and/or national research committee and with the 1964 Declaration of Helsinki and its later amendment.

## Data Availability

Data supporting the findings are available from the corresponding author upon reasonable request.
